# Histological comparison of the lamellar tissue of Iberian origin breed horses created in semi-feral conditions or in an intensive system

**DOI:** 10.1371/journal.pone.0286536

**Published:** 2023-06-01

**Authors:** Bruno Dondoni Malacarne, Rodrigo Ribeiro Martins, Cahuê Francisco Rosa Paz, João Victor Almeida Alves, Lucas Antunes Dias, Marina Alcantara Cavalcante, Alison Miranda Santos, André Guimarães Maciel Silva, Britta Sigrid Leise, Armando Mattos Carvalho, Rafael Resende Faleiros

**Affiliations:** 1 Marion DuPont Equine Medical Center, Virginia Tech, Leesburg, Virginia, United States of America; 2 Equinova Research Group, Universidade Federal de Minas Gerais, Belo Horizonte, Brazil; 3 Centro Universitário de Mineiros-UNIFIMES, Mineiros, Goiás, Brazil; 4 Pontifícia Universidade Católica de Minas Gerais, Belo Horizonte, Minas Gerais, Brazil; 5 Veterinary School, Universidade Federal de Minas Gerais, Belo Horizonte, Brazil; 6 Instituto de Medicina Veterinária, Universidade Federal do Pará, Castanhal, Pará, Brazil; 7 Veterinary Clinical Sciences, Louisiana State University, Baton Rouge, Louisiana, United States of America; Federal University of Ceara: Universidade Federal do Ceara, BRAZIL

## Abstract

Although the external conformation of wild horse hooves has been proposed as an ideal model for domesticated modern horses, histological signs of laminitis have been reported among them. With the hypothesis that the lamellar tissue of horses of Iberian origin raised in semi-feral is healthier than those raised in an intensive management system (stall confinement and high-calorie diet intake), the objective was to compare their lamellar tissues. Lamellar tissue samples were taken from the forelimb hoof of eight domesticated Mangalarga Marchador (MM) horses and from six semi-feral Marajoara (MJ) and Puruca (MP) horses. Primary epidermal lamella (PEL) and secondary epidermal lamella (SEL) were measured (length and width) in several regions, and their shapes were morphologically classified into different types. Breed groups were compared using analysis of variance, followed by Tukey or Dunn tests (*P*<0.05). Early signs of laminitis such as abnormal keratinization of the abaxial primary dermal lamella, tapered tips of the axial PELs, pointed tips of the SEL, nuclei condensation, and abnormal spatial orientation of the secondary epidermal basal cells (SELBC), were seen only in MM horses. MP horses had a greater interface of epidermis/dermis contact than MJ horses and more rounded nuclei in the round SELBC than MM horses. In agreement with the study hypothesis, semi-feral MJ and MP horses’ lamellae were classified as healthier than MM horses, which showed early signs of endocrinopathic laminitis.

## Introduction

The hoof of the horse is a unique structure with a complex anatomy [1] essential for locomotion and athletic performance. Similarities and differences between domesticated and feral horses’ hooves were determined based on morphology reported for wild horses in Wyoming [2, 3]. Results from these studies suggested that feral or wild horses have natural balance and ideal hoof conformation owing to natural wearing that could be followed for domestic horses. However, recent studies conducted on feral horses in New Zealand and Australia have revealed numerous abnormalities in the hoof capsule [4, 5], including histological changes consistent with laminitis [6, 7]. Such findings raised suspicion that even in the wild, these animals were subjected to developing endocrinopathic laminitis from ingestion of plants rich in non-structural carbohydrates (NSCs) [6, 7].

Marajoara (MJ) and Puruca (MP) breeds originated from horses brought by settlers 300 years ago from the Iberian Peninsula to the island of Marajó, Pará State, Brazil [8]. The MJ breed was generated by the miscegenation of these horses with Arab and other purebred horses of Iberian origin, whereas the MP breed was generated by the crossing of MJ horses and Shetland ponies [8]. These horses are raised on native grasses grown in low fertility soils without mineral supplementation [9]. Hooves of these horses do not experience human intervention. They are self-maintained according to geographical and climatic conditions to which they are exposed [10]. It is hypothesised that during the rainy season flooded regions are formed, and hooves become submerged in a water layer and are only slightly worn. With rising temperatures in the summer and transition to the dry season, the soil becomes hard, and the hooves become worn, thus resulting in self-maintaining balance over the seasons [11].

In contrast, another domesticated Iberian Breed, Mangalarga Marchador (MM), undergo routine foot care and are known for their risk of developing laminitis. These horses have been highly bred, selecting for desirable show ring traits, but not necessarily for ideal hoof structure. Additionally, these horses are typically raised in partial stall confinement and on a diet rich in NSCs [12–14].

The aim of this study was to compare the histology of hoof lamellae in Iberian breeds raised in modern domesticated conditions (MM) with those raised in a semi-feral state (MJ and MP). Our hypothesis was that the hoof tissue of horses raised in semi-feral conditions (i.e., exposed to daily exercise, limited access to nutrition, and no protection from environmental and pathogenic agents) would exhibit healthier lamellar tissue than horses of similar origin raised under intensive conditions (i.e., confined to stalls and fed high-energy diets rich in non-structural carbohydrates).

## Materials and methods

### Experimental design

This study was approved by the Ethics Committee on Animal Use at the Universidade Federal de Minas Gerais (UFMG) (approval number: 205/2018). Lamellar biopsies were obtained from three different groups of horses and used for histological evaluation.

### Animals

Twelve semi-feral horses, six each of MJs and MPs, were randomly selected from the herd of Perseverança Ranch, Marajó Island, Soure, Pará State, Brazil. The island is exposed to hot and humid weather, with a mean temperature of 27.0°C, and annually 2.900 mm of rain. The seasons are defined as extremely rainy (December—June) and less rainy, or “dry season” in the rest of the months, and the animal’s diet consists only of native grasses, which grows in soils of poor quality [9]. The main ones are *“Taboquinha” (Panicum zizanoides)*, *“Arroz Bravo”(Oriza spp)*, *“Uamã” (Luziola sptuceana)*,*“Perimembeca” (Paspalum repens)*,*“Canarãna”(Echinochloa polystachya)*, *“Pancuã” (Axonopus affinis)*, *“Andrequice” (Leerxia hexandra Swartz)* [9].

The MJ horses were comprised of four stallions and two geldings with mean (± standard deviation [SD]) weight of 300.83 ± 17.15 kg and age of 3.83 ± 0.98 years. The MP horses were comprised of two stallions and four geldings with mean (± SD) weight of 252.5 ± 21.79 kg and age of 7.75 ± 3.77 years. All horses in both groups had body condition scores between 5 and 6 [15]. Although saddle broke, MJ and MP horses were considered semi-feral, as they had not been ridden or handled in years. Horses were assessed from a distance through static and dynamic inspection. Those that presented any abnormality in conformation or lameness were discarded.

To compare animals with different environmental and management conditions, archived lamellar tissue samples from eight MM horses were used (four non-pregnant mares and four geldings), with an average weight of 402.77±58.31 kg and age of 4±0.41 years, all with a body condition score between 8 and 9 [16]. These horses were confined in stall and received digestible energy (DE) in quantities twice maintenance requirement [17], over 150 days, as described by Ribeiro et al. [18]. Half of the DE was provided as concentrate and half as forage (*Cynodon dactylon (L*.*) Pers*. *Var*. *‘Coast cross’*) at 2% of body weight.

### Lamellar biopsies

The semi-feral horses were placed into a corral and restrained with halters and placed in clean and grassy locations. They were sedated with detomidine (Dormiun V, Agener Uniao) at 40 μg/kg IV; phenylbutazone (Equipalazone, Dechra) 4.4 mg/kg IV, sulfadiazine + trimethoprim (Tridiazin, Vansil) 15 mg/kg IM, and tetanus antitoxin (LemaInjex) 5000 IU IM were administered. A 14-gauge catheter was placed within the jugular vein; the horses were administered ketamine (Cetamin Syntec 10%) 2 mg/kg IV for anaesthesia induction. Total intravenous anaesthesia was maintained by administering a continuous rate infusion of guaifenesin (5%), ketamine (Cetamin Syntec 10%) 2 g, and xylazine 1 g (Equisedan 10%, J.A. Saude animal) diluted in 1 L of lactated ringer solution, at rate of 1.0–1.5 ml/kg/h. Hoof desensitisation was achieved with an abaxial nerve block using 60 mg lidocaine hydrochloride (Xylestesin, Cristalia) per site.

Aseptic preparation of the dorsal hoof wall was performed using 2% chlorhexidine and alcohol 70%. Lamellar biopsies were obtained from the dorsal portion of hoof capsule [19], and the samples were placed in 10% buffered formalin. Subsequently, the biopsy site was covered with dental impression material (Perfil Coltene, Brazil) soaked in 10% gentamicin, followed by application of a compressive bandage. The surgical wound was cleaned with lactated Ringer’s solution followed by dressing. Any horse demonstrating lameness in the postoperative period was administered phenylbutazone 4.4 mg/kg IV every 24 h for 5 days.

The same biopsy technique was used on the MM horses; however, they were restrained into stocks and the procedure was performed in standing position under sedation (detomidine) and perineural anaesthesia (lidocaine) of the digital nerves.

### Histological assessment

After inclusion in paraffin, tissue samples from the three groups were cut into 5 μm slices and stained with periodic acid-Schiff and haematoxylin and eosin methods. The histological images were digitised at the *Centro de Aquisição e Processamento de Imagem*. Histological evaluation was performed using Panoramic Viewer 8.0 and Image J.

Histomorphometric assessment of lamellae was performed as previously described [20]. At 5× magnification (500 μm), primary epidermal lamellae (PEL) length was measured from abaxial (closest to hoof wall) to axial regions (adjacent to DP). PEL width was measured in three sections: abaxial (within initial 10% of PEL length closest to hoof capsule), intermediate (middle 50% of PEL length), and axial (within last 90% of PEL length closest to DP). Ten randomly selected fields were evaluated from each sample. PEL length and width were obtained and averages of each of the structures were measured. Secondary epidermal lamellae (SEL) length and width were measured at 20× magnification (100 μm) in the abaxial, intermediate, and axial sections of the PEL, as described above. Thirty SELs per PEL were selected per histological sample i.e., 10 in each region (five on the right side and five on the left side of the keratinized axis). Angles formed between the PEL and SEL were measured at each location. The distance between the end of the keratinized axis and axial tip of the PEL [21], and random tubular density on the stratum medium in a total area of 1300 × 200 μm was also determined.

Histological assessment of axial ends of the PEL were classified as standard, elongated, sharp, or bifurcated [22]. On the abaxial portion, the border of the PDL was classified similarly to the axial ends of PEL, with addition of proliferative, separated and keratinized shapes [22]. Degree of abaxial keratinization was also graded as normal, slightly increased (1–10 extra layers), moderately increased (10–30 layers), or markedly increased (> 30 layers). Amount of epidermal tissue bridging in a PDL was defined as none, mild (1–2 epithelial bridges), or moderate (> 2 epithelial bridges) [22]. The presence of epithelial islands (isolated round or ovoid epithelial structures adjacent) and lakes (small or large cavities that were either empty or filled with homogeneous eosinophilic proteinaceous material) were also evaluated and recorded if present [22]. Ten randomly selected SELs were classified as standard, tapered, club-shaped, bifurcated, fused, or separated [22]. The presence of SEL keratinization or hyperplasia of the suprabasal layer was also recorded. The percentage of basal epidermal cells with fusiform or rounded nuclei was determined by classifying 100 randomly selected nuclei in 10 microscopic fields observed at 40× magnification.

### Statistical analysis

The normality of the data distribution was checked using the Shapiro–Wilk test. Normally distributed variables were subjected to analysis of variance (ANOVA), followed by Tukey’s test for comparison between groups. Non-normal data underwent logarithmic transformation, and normal data were analysed as described. When normality was not obtained by transformation, the data were analysed using the Kruskal-Wallis test, followed by Dunn’s test. GraphPad Prism 7.0 (GraphPad Software, La Jolla, California, USA) was used for statistical analysis. Statistical significance was set at *P*<0.05.

## Results

### Lamellar histomorphometry

As shown in Table 1, the length of the PEL was greater in MP and MM animals than in MJ animals (*P* = 0.0032 and *P* = 0.0001, respectively). Distance between the keratinized axis and axial tip of the PEL was greater in MJ horses than in MP horses (*P* = 0.0007), but there was no difference in relation to MM (*P* = 0.9999).

**Table 1:** Means (± SD) of the histological measurements of hoof lamellar tissue of the Marajoara (MJ), Puruca (MP) and Mangalarga Marchador (MM) breeds.

General measurements	Breed		
	MJ	MP	MM
**PEL length^#^ (μm)**	2430±398.1^b^	2643±333.8^a^	2759±257.4^a^
**Kera-axial tip axis^#^ (μm)**	556±204^a^	434.6±120.4^b^	517±119.8^a^
Abaxial PEL width* (μm)	248.9±38^ab^	299.5±43.08^a^	230.6±28.84^b^
Interm. PEL width* (μm)	211±21.38^b^	262±35.59^a^	168.6±33.41^b^
Axial PEL width* (μm)	190.4±19.07^a^	219±25.62^a^	140.1±28.05^b^
Total PEL width* (μm)	216.7±35.87^b^	260.2±47.52^a^	179.8±48.17^c^
**Abaxial angle PEL/SEL^#^ (°)**	40.11°±8.7^b^	41.81°±10.44^b^	59.16°±16.12^a^
**Interm. angle PEL/SEL^#^ (°)**	43.41±10.73^a^	44.29±12.93^a^	42.44±23.54^a^
**Axial angle PEL/SEL^#^ (°)**	81.95±21.63^a^	71.63±24.63^ab^	61.6±34.62^b^
**SEL**			
Abaxial SEL length* (μm)	127.7±26^b^	157.8±33.41^a^	95.64±23.04^c^
Abaxial SEL width* (μm)	17.65 ±3.54^b^	20.09±2.71^a^	18.91±3.68^ab^
**Interm. SEL length^#^ (μm)**	142.8±42.64^a^	161.8±59.14^a^	110.9±56.36^b^
**Interm. SEL width^#^ (μm)**	21.86±4.63^b^	25.43±4.39^a^	20.79±4.27^b^
**Axial SEL length^#^ (μm)**	95.32±28.64^a^	96.9±55.72^a^	71.02±25.73^b^
Axial SEL width* (μm)	32.76±7.42^a^	33.25±7.19^a^	23.74±5.61^b^
Tubules n°/area*	22.17±2.78^a^	23.17±7.73^a^	22.13±4.94^a^
SELBCs nuclei* (%)	16.05^ab^	19.8^a^	8.80^b^

PEL, primary epidermal lamina; SEL, secondary epidermal lamina; SELBCs, secondary epithelial lamellae basal cells; Interm, intermediate; Kera, keratinized; SD, standard deviation.

^#^Data submitted to the Kruskal-Wallis tests followed by Dunn.

*Data submitted to the Tukey test.

Means followed by the same letters do not differ between lines (P <0.05).

Width of the PEL in the abaxial region was greater in MP than in MM (*P* = 0.0101). In the intermediate region, MP showed greater width than MJ (*P* = 0.0287) and MM (*P* = 0.0002). In the axial portion, the width was greater both in the MP (*P*≥0.0001) and in MJ *(P* = 0.0026) than in MM. Total assessment of the PEL width indicated that the three breeds differed from each other in three different regions. MPs had a wider PEL than MJ (*P* = 0.0134) and MM (*P*<0.0001), whereas MJ had a wider PEL than MM (*P* = 0.0325).

The angle formed between the PEL and SEL in the abaxial region of MM breed was greater than that of MP (*P* = 0.0001) and MJ (*P*≥0.0001). Angles were similar in intermediate regions. However, in the axial region, MJ horses had a significantly greater angle than MM horses (*P* = 0.0160).

Evaluation of SEL in the abaxial region revealed that the length was significantly different among the three breeds. SELs of MP were longer than those of MJ (*P* = 0.0002) and MM (P≤0.0001), and SELs of MJ were longer than those of MM (*P*<0.0001). SEL width in this region was greater in MP than in MJ (*P* = 0.0166). In the intermediate region, MM had shorter SEL length than MJ (*P* = 0.0142) and MP (*P* = 0.0003); SEL width was greater in MP than in MM (*P* = 0.0002) and MJ (*P* = 0.0132). SEL was shorter in the axial region in MM than in MJ (*P* = 0.0009) and MP (*P* = 0.0462); moreover, SEL width of MM was smaller than MJ (*P*≤0.0001) and MP (*P*≤0.0001).

**Fig 1:**
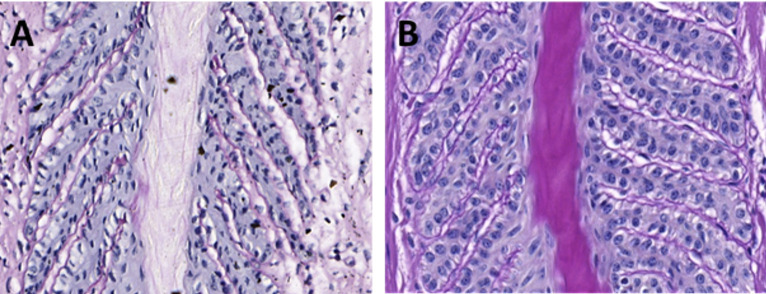
Photomicrographs of the equine hoof secondary epidermal lamellae (SEL) of the equine (20x magnification (100 μm), and periodic acid-Schiff staining). Note the predominance of SEL with a tapered shape and basal epithelial cells with condensed nuclei in the biopsy taken from a Mangalarga Marchador breed horse **(A)** compared with the predominance of rounded and not condensed nuclei in the basal epithelial in a biopsy taken from Puruca breed horse **(B)**.

Evaluation of the nuclei of the secondary epidermal lamellae basal cells (SELBC) revealed that MP (Fig 1B), had a higher rate of rounded nuclei than MM (*P* = 0.0039) (Fig 1A). MJs presented twice the percentage (16.5%) compared to MM (8.80%), but with *P* = 0.0552. Notably, in the axial region of the PEL, MM presented more condensed nuclei and disorientation regarding angulation with the basement membrane.

### Lamellar histomorphology

**Fig 2:**
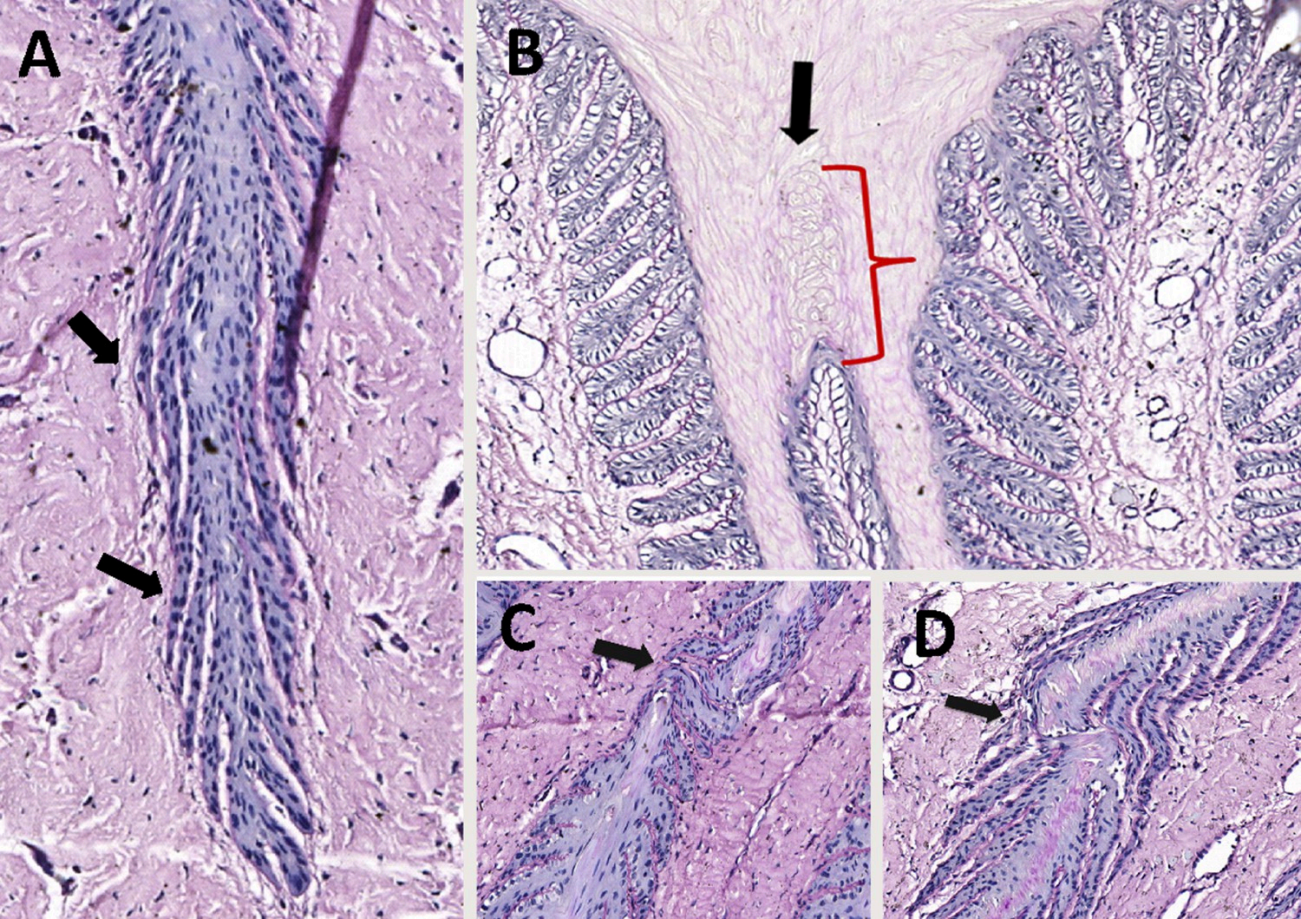
Photomicrograph of the hoof lamellar tissue from Mangalarga Marchador (MM) horses (10x magnification (200 μm), periodic acid-Schiff staining). **(A)** An axial portion of a primary epidermal lamina (PEL) is observed, with secondary epidermal lamellae (SEL) of tapered shape (arrows), with a predominance of basal epithelial cells with condensed nuclei and missing the perpendicular orientation to the basal membrane. **(B)** Note unusual proliferation and cornification of epidermal epithelial tissue at the abaxial border of a primary dermal lamina (arrow) **(C and D)** Areas of discontinuity are observed in the primary epidermal lamellae (arrows).

MM horses were the only ones that showed signs of fragility in lamellar tissue, characterised by the occurrence of abnormal keratinization of the PDL border (Fig 2B), discontinuity of PEL (Fig 2C and 2D), SEL with tapered shape (mainly in the axial region) (Fig 2A), and abnormal changes in SELBC, such as nuclei condensation and loss of perpendicular angle to the basal membrane (BM) (Fig 2A).

No significant difference existed in the morphology of the axial tip of the PEL between breeds. However, at the abaxial end of the PDL, MM horses showed a higher percentage of sharp shapes than MJ (*P* = 0.0426) and MP horses (*P* = 0.0426). MM was also the only one to show PDL in a keratinized format, different from MJ (*P* = 0.0150) and MP (*P* = 0.0150). Bifurcated shape was significantly higher in MJ than in MM (*P* = 0.0419), Table 2.

**Table 2:** Means (± SD) of the percentages of occurrence of different types of extremities in the primary epidermal layers (PEL) and primary dermal layers (PDL) as previously reported [22] of the lamellar tissue of horses, of the Marajoara (MJ), Puruca (MP) and Mangalarga Marchador (MM) breeds.

Morphology		PEL (%)	
	MJ	MP	MM
**Standard** ^**#**^	71.6±9.8^a^	66.6±17.5^a^	54.28±19.8^a^
**Sharp** ^**#**^	25±5.4^a^	21.66±7.5^a^	27.14±13.8^a^
**Tapered** ^**#**^	1.6±4^a^	6.6±12.1^a^	17.14±14.9^a^
**Bifurcated** ^**#**^	1.6±4^a^	5±8.3^a^	1.4±3.7^a^
**PDL**			
**Standard** ^**#**^	86.66±15^a^	93.33±12.1^a^	83.33±12.7^a^
**Sharp** ^**#**^	0^b^	0^b^	5±5.3^a^
**Bifurcated** ^**#**^	13.33±15^a^	6.66±12.1^ab^	1.66±3.7^b^
**Keratinized** ^**#**^	0^b^	0^b^	11.66±8.9^a^
**Proliferative** ^**#**^	0^a^	0^a^	0^a^
Separated^#^	**0a**	**0a**	**0a**

SD, standard deviation.

^#^Data submitted to the Kruskal-Wallis tests followed by Dunn.

*Data submitted to the Tukey test.

Means followed by the same letters do not differ between lines (P <0.05).

No difference existed in the lamellar morphology of the SEL between breeds in the abaxial region, with the standard format predominating. The standard format was predominant in the intermediate region; however, it was significantly smaller in MM than in MP (*P* = 0.0148). MM horses had a greater number of tapered shapes within the SEL than MP horses (*P* = 0.0251). In the axial portion of the PEL, only MM presented tapered SEL compared to MJ and MP (*P* = 0.0449). MJ horses showed a significantly greater number of club shape SELs than MM horses in the axial portion (*P* = 0.0450). Bifurcated format was more prevalent in MP than in MM (*P* = 0.0262), Table 3.

**Table 3:** Means (± SD) of the percentages of occurrence of different types of extremities in the secondary epidermal lamellae (SEL) of the lamellar tissue of horses, of the Marajoara (MJ), Puruca (MP) and Mangalarga Marchador (MM) breeds.

Morphology SEL	Abaxial region (%)		
	MJ	MP	MM
Standard*	85±6.5^a^	93±5.6^a^	80.33±12.3^a^
**Tapered^#^**	4.33±4.2^a^	3±6.4^a^	10±11.8^a^
**Club head^#^**	2.6±3.9^a^	0.6±1^a^	1±1.9^a^
**Hyperplasic SBL ^#^**	0.33±0.8^a^	0^a^	0.33±0.7^a^
**Bifurcated^#^**	4.3±2.6^a^	3.3±3.2^a^	6±6.9 ^a^
**Fused^#^**	0^a^	0^a^	0^a^
**Separated^#^**	0^a^	0^a^	0^a^
**Keratinized ^#^**	0^a^	0^a^	0^a^
	**Intermediate region (%)**		
**Standard^#^**	85±8.4^ab^	89±7.6^a^	62.66±19.4^b^
**Tapered^#^**	6.66±6.1^ab^	2±2.5^b^	23.42±20.^a^
**Club head^#^**	5.33 ±4.5^a^	4.33±7 ^a^	3.66±4.7 ^a^
**Hyperplasic SBL^#^**	1.66±2.6 ^a^	3.66±4.2 ^a^	4±6.9 ^a^
**Bifurcated^#^**	1.33±2 ^a^	1±1 ^a^	4±5.5^a^
**Fused^#^**	0 ^a^	0^a^	0 ^a^
**Separated^#^**	0^a^	0^a^	0^a^
**Keratinized^#^**	0^a^	0^a^	0^a^
	**Axial region (%)**		
**Standard^#^**	64.33±15.2^a^	63±8.4^a^	74.33±7.5^a^
**Tapered^#^**	0^b^	0^b^	7.71±7.5^a^
**Club head^#^**	12.33±7.7 ^a^	8.33±5.5 ^ab^	2.33±1.78^b^
Hyperplasic SBL*	20.33±11.8^a^	19.66±6.3^a^	14.66±6.1^a^
Bifurcated*	3.33±3.7 ^ab^	9±6.2 ^a^	1.66±2.3^b^
**Fused^#^**	0^a^	0^a^	0.66±1.5^a^
**Separated^#^**	0^a^	0^a^	0^a^
**Keratinized ^#^**	0^a^	0^a^	0^a^

SBL, suprabasal layer; SD, standard deviation.

^#^Data submitted to the Kruskal-Wallis tests followed by Dunn.

*Data submitted to the Tukey test.

Means followed by the same letters do not differ between lines (P <0.05).

## Discussion

The present study demonstrates that semi-feral horses, despite having histological variations related to the environment, nutrition, and hoof self-maintenance process, presented healthier lamellar tissue compared to horses raised in intensive systems.

The MM horses that received high concentrations of NSCs had lesions in the lamellar tissue, unsurprising since hormonal imbalances were previously identified [16, 18, 23, 24]. The lesions included abnormal keratinization of the abaxial border of several PDLs, PEL discontinuities, and condensed nuclei of SELBCs with loss of their orientation perpendicular to the BM. These findings were consistent with the initial stage of laminitis, with lesions classically described in natural cases associated with hyperinsulinaemia [22], and in models of insulin-induced laminitis [25].

The length of SEL was shorter in MM in all regions of the PEL when compared with the other groups, contradicting the results found in natural cases of hyperinsulinaemia [22], and in models of insulin [21, 25] or oligofructose [21] inducted laminitis, where there is stretching of the SEL. Since MM horses in this study were in the early stages of insulin dysregulation [23], it is believed that the shortening of SEL extent is possibly associated with the process of sinking DP [16], which may precede the characteristic stretching seen in horses artificially exposed to much higher blood insulin concentrations [21, 25] and in horses in more advanced stages of hyperinsulinaemia, which already have clinical evidence of laminitis [22].

Changes in the morphology of SEL are associated with early stages of laminitis [26]. Considering that MM horses presented a shortening of the SEL, many of them presented a tapered morphology, with or without elongation. Tapered SEL were found only axially in MM, with a higher percentage in the intermediate region compared to MP. It can be hypothesised that this is the initial stage for the collapse of the basement membrane at the SEL tips since it is considered the focus of injury in carbohydrate overload laminitis models [26]. In contrast, BM lesions are considered minimal in models of endocrinopathic laminitis [27].

Induction of laminitis due to insulin infusion and oligofructose overload revealed a greater distance between the keratinized axis of the PEL and its axial tip in comparison to healthy animals [21]. However, analysing the data from the present study, MJ horses (without any sign of laminitis) had a similar distance to the MM horses measuring 556 μm and 517 μm, respectively. Therefore, caution is advised when interpreting the value of this distance in the early stages of disease, since it is not as significant as in induction models or more advanced stages of laminitis.

The marked occurrence of SELBC with round nuclei in semi-feral horses is worth noting. MP horses had a significantly higher percentage of nuclei with rounded shapes than MM. Also, although there were no statistically significant differences, the MJ group presented almost twice as many nuclei with rounded shapes as the MM group. According to Pollit [28], the SELBC nuclei naturally have an ovoid shape, and one of the first findings in horses subjected to laminitis due to carbohydrate overload was the change in their morphology to a round shape [26, 29]. However, it has been stated that the round shape of SELBC nuclei is not specific to laminitis and can be found in horses without any signs of disease [25].

Changes in the appearance of the cell nuclei can occur pathologically when the cell suffers temporary or permanent damage that eventually induces cell death or physiologically, when the cell adapts to different causes, such as simple nutritional changes or changes in mechanical load [30]. Another reason is diet variation during the year, as seasons can affect the nutritional quality of forages [31]. Thus, these animals adapt to low nutrient availability because of an absence of supplementation during periods of deficiency. Therefore, it is speculated that rounded nuclei in SELBC may be the result of genetic transcription in semi-feral horses that require greater cellular adaptation to environmental and nutritional conditions, and the process of hoof self-maintenance.

Other differences in the hoof lamella between feral and domesticated horses have previously been described, revealing that feral horses’ fetuses had a higher density of PEL [32]. Interestingly, in our study, at the ends of the PDLs (abaxial), the bifurcated pattern was significantly more prominent in MJ than in MM. In MP horses, this characteristic was more evident in the axial region of the SEL, having a higher percentage of bifurcation compared to MM horses. PDL bifurcation has been associated with the growth of PELs [33] and can be found in all ages [34]. This shape may represent a potential mechanism for increasing lamellar surface area and weight support; yet it only represents a small amount of the lamellar tissue [35]. Therefore, these differences may be the result of adaptation of the hoof to environmental conditions and mechanical loads applied to the lamellar tissue in semi-wild horses, evaluated in this study.

It is important to consider several undetermined factors when interpreting the present results, including but not limited to the small sample size, differences in age, sex, and breed between the groups, as well as the distinct geographic conditions. However, this is the first study to demonstrate that semi-feral horses have lamellar histological characteristics that are very different from those observed in horses confined and excessively fed, which is a common practice in the modern Brazilian horse management system. Further studies with larger sample sizes are needed to describe the histological patterns of semi-feral horses.

## Conclusions

Our preliminary findings suggest that Marajoara and Puruca horses from Marajó Island raised in semi-feral conditions, with daily exercise, limited access to nutrition and no protection to environmental and infectious factors, have healthier hoof lamellar characteristics than Mangalarga Marchador horses raised under modern domesticated conditions characterized by stall confinement and overfeeding practices. Despite their smaller stature, MP horses have greater length and width of their primary and secondary epidermal lamellae than other breeds. Conversely, several abnormal findings observed in MM horses indicate the detrimental effects of management practices on equine hoof health.

## Supporting information

S1 Data (ZIP)

## References

[1] Pollitt CC. Anatomy and physiology of the inner hoof wall. Clinical Techniques in Equine Practice. 2004; 3: 3–21.

[2] Ovnicek G, Erfle J, Peters D. Wild horse hoof patterns offer a formula for preventing and treating lameness. 41^th^ Proceedings of the American Association of Equine Practitioners, Lexington, KY, USA, 3th-6th December. 1995; pp. 258–260.

[3] Ovnicek GD, Page BT, Trotter GW. Natural balance trimming and shoeing: its theory and application. Veterinary Clinics of North America. Equine Practice. 2003: 19; 353–77. 14575164 10.1016/s0749-0739(03)00017-8

[4] Hampson BA, De Laat MA, Mills PC, Pollitt CC. The feral horse foot. Part A: observational study of the effect of environment on the morphometrics of the feet of 100 Australian feral horses. Australian Veterinary Journal. 2013; 91: 14–22. doi: 10.1111/j.1751-0813.2012.00995.x 23356367

[5] Hampson BA, Ramsey G, Macintosh AMH, Mills PC, De Laat MA, Pollitt CC. Morphometry and abnormalities of the feet of Kaimanawa feral horses in New Zealand. Australian Veterinary Journal. 2010; 88: 124–131. 20402699 10.1111/j.1751-0813.2010.00554.x

[6] Hampson BA, De Laat MA, Beausac C, Rovel T, Pollitt CC. Histopathological examination of chronic laminitis in Kaimanawa feral horses of New Zealand. New Zealand Veterinary Journal. 2012; 60: 285–289. doi: 10.1080/00480169.2012.682271 22621688

[7] Hampson BA, De Laat MA, Mills PC, Walsh DM, Pollitt CC. The feral horse foot. Part B: radiographic, gross visual and histopathological parameters of foot health in 100 Australian feral horses. Australian Veterinary Journal. 2013; 91: 23–30. doi: 10.1111/avj.12017 23356368

[8] Costa MRTR. A História dos Eqüinos na Amazônia: ênfase ao cavalo marajoara. 2008. https://www.embrapa.br/busca-de-publicacoes/-/publicacao/409962/a-historia-dos-equinos-na-amazonia-enfase-ao-cavalo-marajoar*a* (accessed 10 July 2019).

[9] Embrapa. Séries sistemas de produção. Boletim. Sistemas de produção de gado de Corte, Soure-Ilha de Marajó-PA. 1976. https://ainfo.cnptia.embrapa.br/digital/bitstream/item/44240/1/SID-DOCUMENTOS-14-SISTEMAS-DE-PRODUCAO-PARA-GADO-DE-CORTE-SOURE-ILHA-DO-MARAJO-PA-CDU-636-2338.pdf (accessed 12 July 2019)

[10] Florence L, Mcdonnell SM. Hoof growth and wear of semi-feral ponies during an annual summer ‘self-trimming’ period. Equine Veterinary Journal. 2006; 38: 642–645. doi: 10.2746/042516406x158350 17228579

[11] Malacarne BD. Estudo descritivo e comparativo, morfológico, radiológico e histológico dos cascos dos equinos das raças Marajoara e Puruca. Dissertação de mestrado na área de clínica e cirurgia animal, Universidade Federal de Minas Gerais, Brasil. 2020.

[12] Rezende ASC, Sampaio IBM, Legorreta GL, Moreira DCA. Effect of two different nutritional programs on orthopedic alterations in Mangalarga Marchador foals. J. Equine Vet. Sci., 20 (2000), pp. 651–656

[13] Inácio DFS, Rezende ASC, Saliba EOS, Silva RHP, Maruch S, Lana AMQ, et al. Dry Matter Intake and Apparent Digestibility of Nutrients in the Ration of Mangalarga Marchador Weanling Horses Fed Sorghum Silage Versus Grass Hay, Journal of Equine Veterinary Science, v.49, p. 87–91, 2017. 10.1016/j.jevs.2016.09.011.

[14] Magalhães JF, Lima LR, Paz CFR, Rocha Junior SS, Oliveira APL, Duarte PC, et al. 2017. [Spatial relationship between the distal phalanx and the hoof capsule in young Campolina mares with and without obesity.] Relação espacial entre a falange distal e o estojo córneo em éguas Campolinas jovens com e sem sinais de obesidade. Pesquisa Veterinária Brasileira 37(9):1025–1031.

[15] Henneke DR, Potter GD, Kreider JL. Body condition during pregnancy and lactation and reproductive efficiency of mares. Theriogenology. 1983; v. 21: p. 897–909.

[16] Ribeiro RM. Relação entre obesidade induzida e laminite endocrinopática em equinos Mangalarga Marchador: aspectos clínicos, laboratoriais, morfométricos e patológicos. Tese de doutorado na área de clínica e cirurgia animal- Universidade Federal de Minas Gerais, Brasil. 2017.

[17] National Research Council, NRC. Nutrient requirements of horses. Education Review Washington, *DC*. National Academies. 2007; 6: pp-360.

[18] Ribeiro RM, Ribeiro DSF, Paz CFR, Gobesso AAO, Faleiros RR. Adiposity and weight gain in Mangalarga Marchador horses subjected to hypercaloric diet. Pesquisa Veterinária Brasileira. 2020; 40: 170–175.

[19] Ribeiro RM, Mendes HMF, Valadares RC, Paz CFR, de Oliveira APL, Junior OS, et al. A novel equine hoof lamellar tissue biopsy technique. Journal of Equine Veterinary Science. 2017: 49: 63–68.

[20] Karikoski NP, Patterson-Kane JC, Asplin KE, McGowan TW, McNutt M, Singer ER, et al. Morphological and cellular changes in secondary epidermal laminae of horses with insulin-induced laminitis. American Journal of Veterinary Research. 2014; 75: 161–168. doi: 10.2460/ajvr.75.2.161 24471752

[21] de Laat MA, van Eps AW, Mcgowan CM, Sillence MN, Pollitt CC. Equine laminitis: comparative histopathology 48 hours after experimental induction with insulin or alimentary oligofructose in Standardbred horses. Journal of Comparative Pathology. 2011; 145: 399–409. doi: 10.1016/j.jcpa.2011.02.001 21429503

[22] Karikoski NP, Mcgowan CM, Singer ER, Asplin KE, Tulamo RM, Patterson-Kane JC. Pathology of natural cases of equine endocrinopathic laminitis associated with hyperinsulinemia. Veterinary Pathology. 2015; 52: 945–956. doi: 10.1177/0300985814549212 25232034

[23] Ribeiro RM, Ribeiro DSF, Cota LO, Leme FO, Carvalho AM, Faleiros RR. Changes in metabolic and physiological biomarkers in Mangalarga Marchador horses with induced obesity. Veterinary Journal. 2021; 270: 105627. doi: 10.1016/j.tvjl.2021.105627 33641803

[24] Ribeiro RM, Ribeiro DSF, Paz CFR, Gobesso AAO, Faleiros RR. Insulin dysregulation in horses with induced obesity. Pesquisa Veterinária Brasileira. 2020; 40: 39–45.

[25] Asplin KE, Patterson-Kane JC, Sillence MN, Pollitt CC, Mc Gowan CM. Histopathology of insulin-induced laminitis in ponies. Equine Veterinary Journal. 2010; 42: 700–706. doi: 10.1111/j.2042-3306.2010.00111.x 21039799

[26] Pollitt CC. Basement membrane pathology: a feature of acute equine Laminitis. Equine Veterinary Journal. 1996; 28: 38–46. doi: 10.1111/j.2042-3306.1996.tb01588.x 8565952

[27] Patterson-Kane JC, Karikoski NP, Mcgowan CM. Paradigm shifts in understanding equine laminitis. Veterinary Journal. 2018; 231: 33–40. doi: 10.1016/j.tvjl.2017.11.011 29429485

[28] Pollitt CC. Foot Structure and function. In: The Illustrated horse’s foot a comprehensive guide, 1^th^ ed. Elsiever Saunders, Louis St, MO USA, 2016; pp. 10–80.

[29] Faleiros RR, Johnson PJ, Nuovo GJ, Messer NT, Black SJ, Belknap JK. Laminar leukocyte accumulation in horses with carbohydrate overload-induced laminitis. Journal of Veterinary Internal Medicine. 2011; 25: 107–115. doi: 10.1111/j.1939-1676.2010.0650.x 21143304

[30] Myers RK, Mcgavin MD, Zachary JF. Cellular adaptations, injury, and death: morphologic, biochemical, and genetic bases. In: Zachary JF, McGavin MD editors, Pathologic basis of veterinary disease. 5^th^ ed. Saunders Elsiever, St Louis, MO, USA. 2013; pp. 7–20.

[31] Fernandes GA. Valor nutritivo do pasto de Urochloa brizantha cv. Marandu em diferentes épocas do ano. Dissertação de mestrado na área de nutrição e produção, Universidade Federal do Mato Grosso, Cuiabá, Brasil. 2016.

[32] Hampson BA, Studies MA, de Laat MA, Mills PC, Pollitt CC. Evaluation of primary epidermal lamellar density in the forefeet of near-term fetal Australian feral and domesticated horses. American Journal of Veterinary Research. 2011; 72: 871–876. doi: 10.2460/ajvr.72.7.871 21728846

[33] Bidwell LA, Bowker RM. Evaluation of changes in architecture of the stratum internum of the hoof wall from fetal, newborn, and yearling horses. American Journal of Veterinary Research. 2006; 67: 1947–1955. doi: 10.2460/ajvr.67.12.1947 17144792

[34] Kawasako K, Higashi T, Nakaji Y, Komine M, Hirayama K, Matsuda K, et al. Histologic evaluation of the diversity of epidermal laminae in hooves of horses without clinical signs of laminitis. American Journal of Veterinary Research. 2009; 70: 186–193. doi: 10.2460/ajvr.70.2.186 19231949

[35] Bowker RM. The growth and adaptive capabilities of the hoof wall and sole: functional changes in response to stress. 49^th^ Annual Convention of the American Association of Equine Practitioners, New Orleans, USA, 21 ^th^—25 ^th^ November. 2003; 146–168.

