# Supplementary figures and images for: Histological comparison of the lamellar tissue of Iberian origin breed horses created in semi-feral conditions or in an intensive system
Source: PLoS One. 2023 Jun 1;18(6):e0286536. doi: 10.1371/journal.pone.0286536 (PMC10234555; doi:10.1371/journal.pone.0286536)

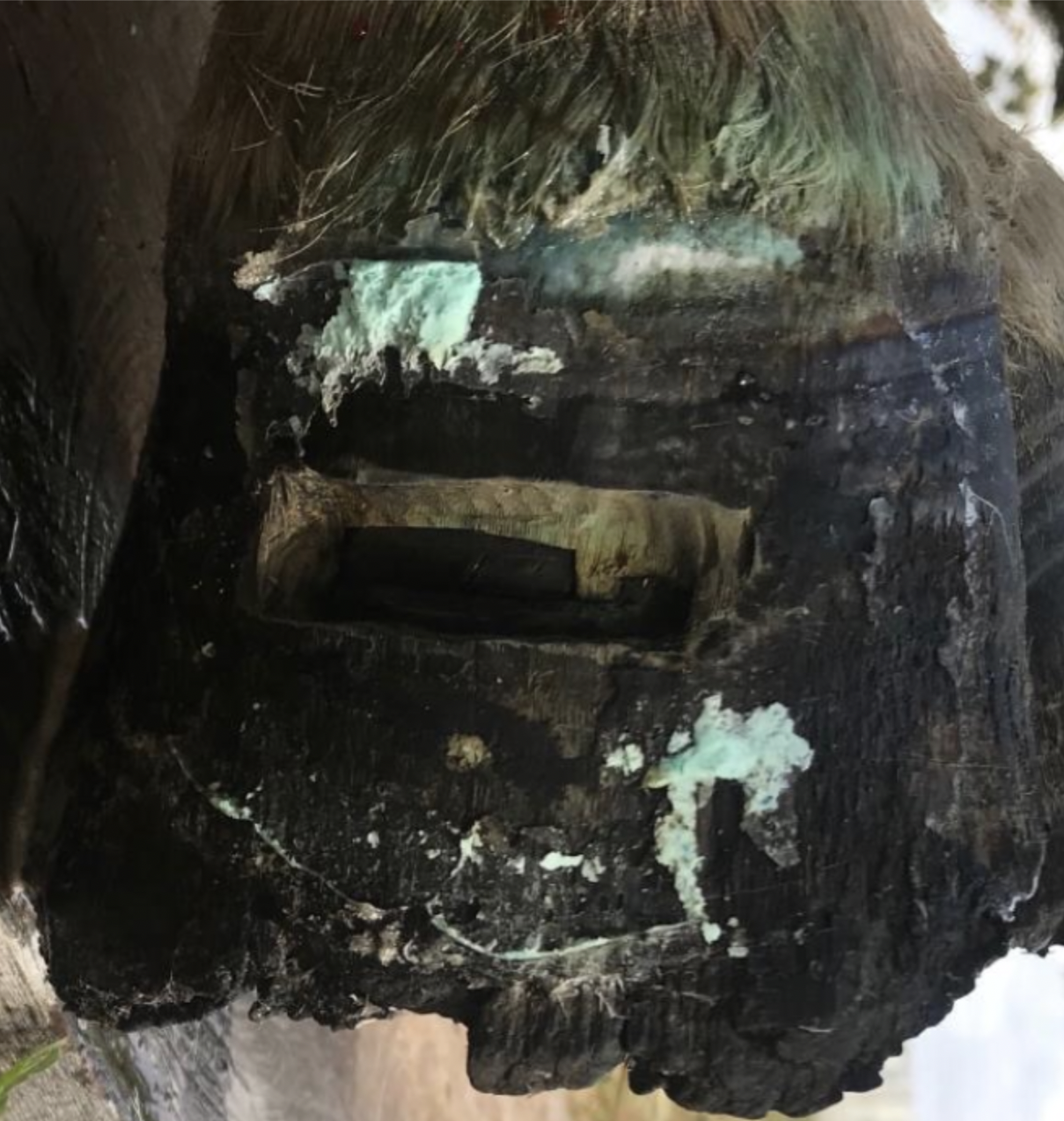

Supplement: S1 Data — (ZIP) [file pone.0286536.s001.zip › MJ hoof 3 days post byopsy.tif]

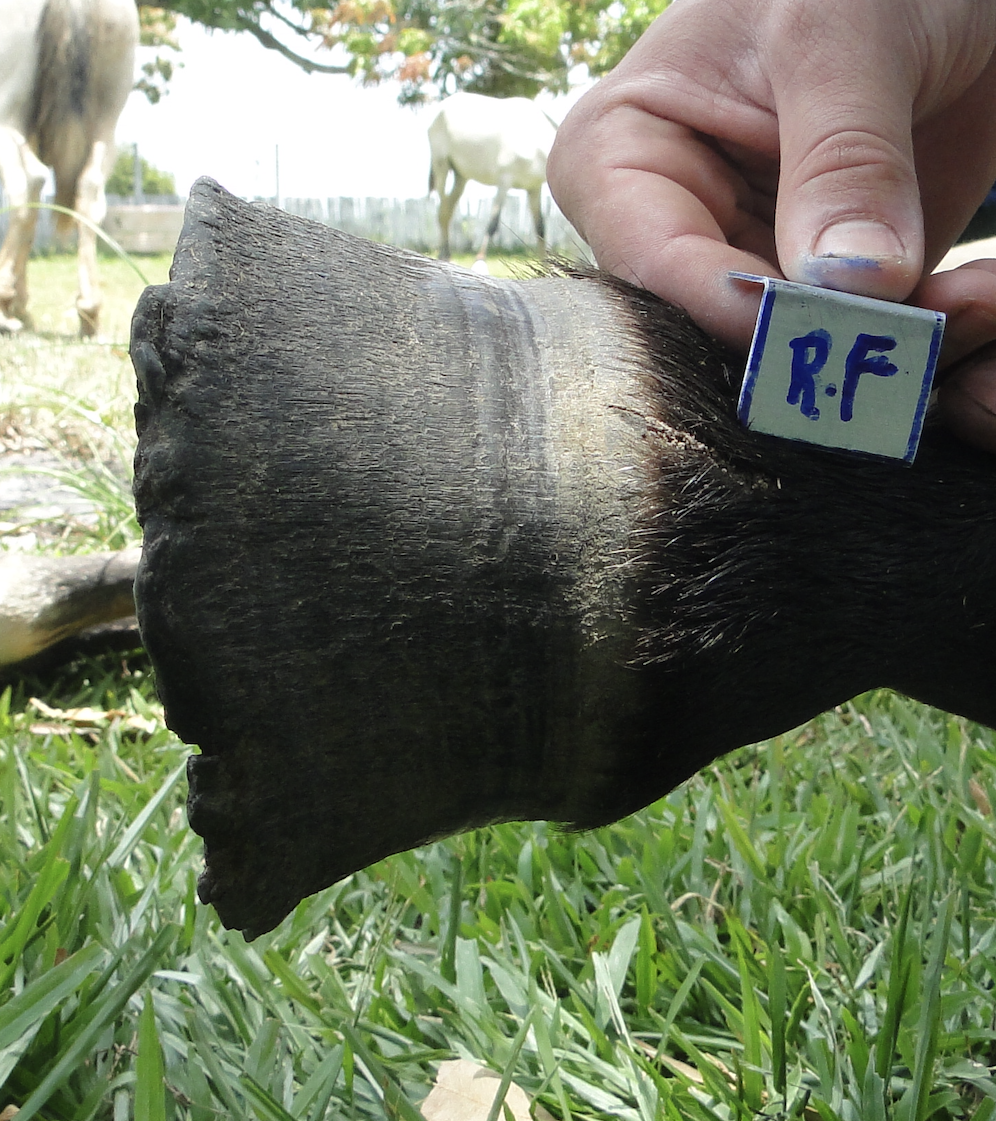

Supplement: S1 Data — (ZIP) [file pone.0286536.s001.zip › MJ hoof pre byopsy.tif]

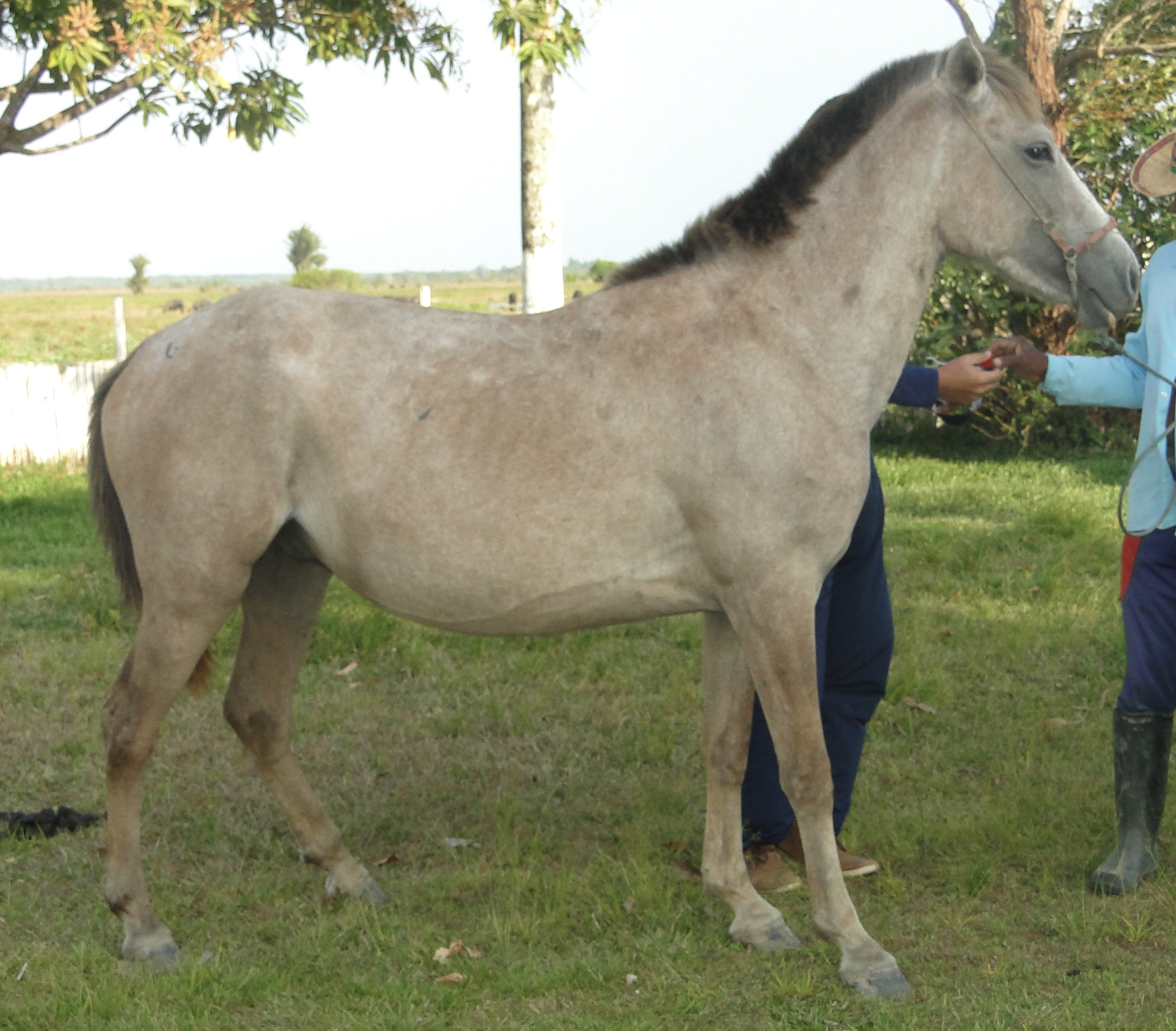

Supplement: S1 Data — (ZIP) [file pone.0286536.s001.zip › MJ horse.tif]

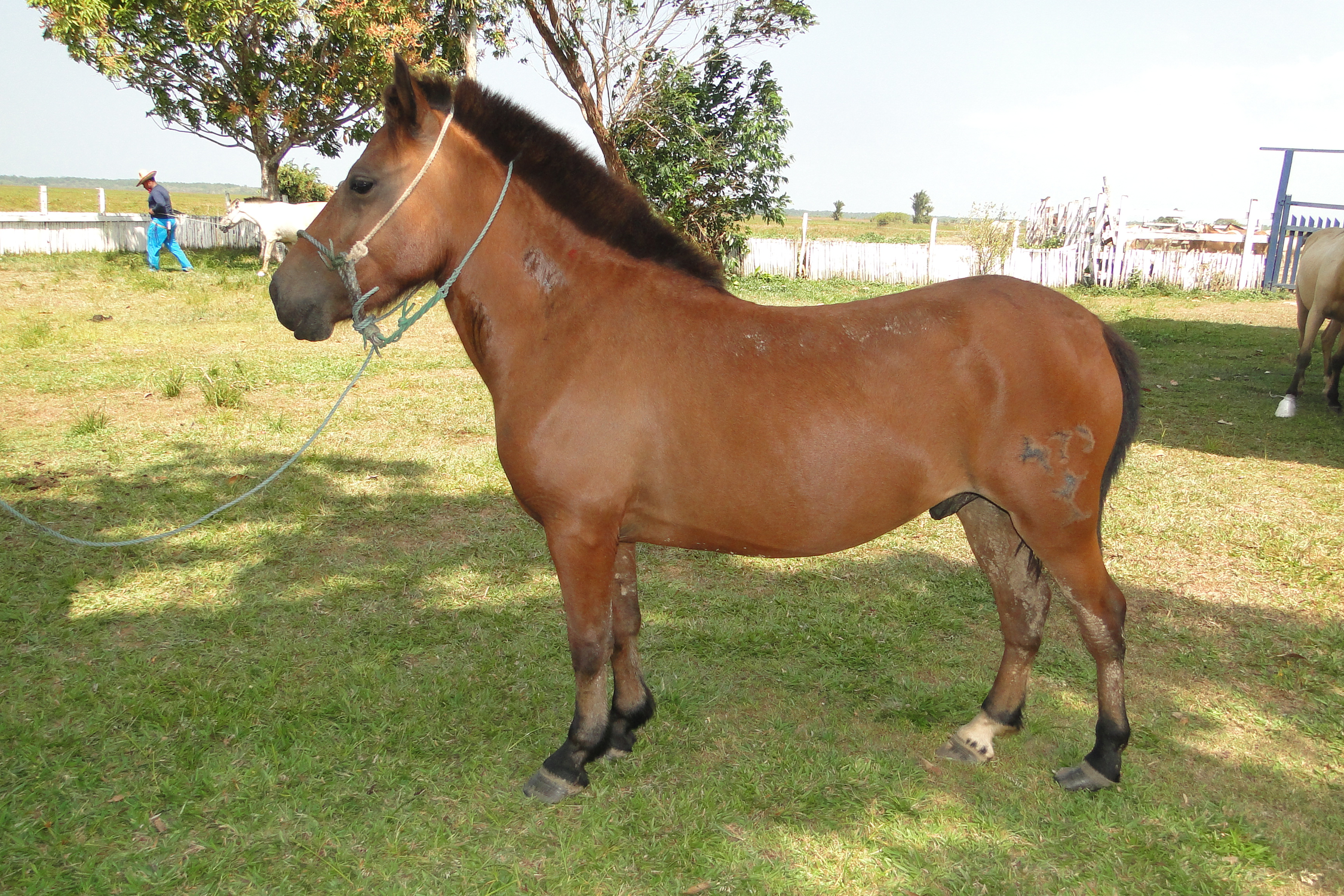

Supplement: S1 Data — (ZIP) [file pone.0286536.s001.zip › MP horse.tif]

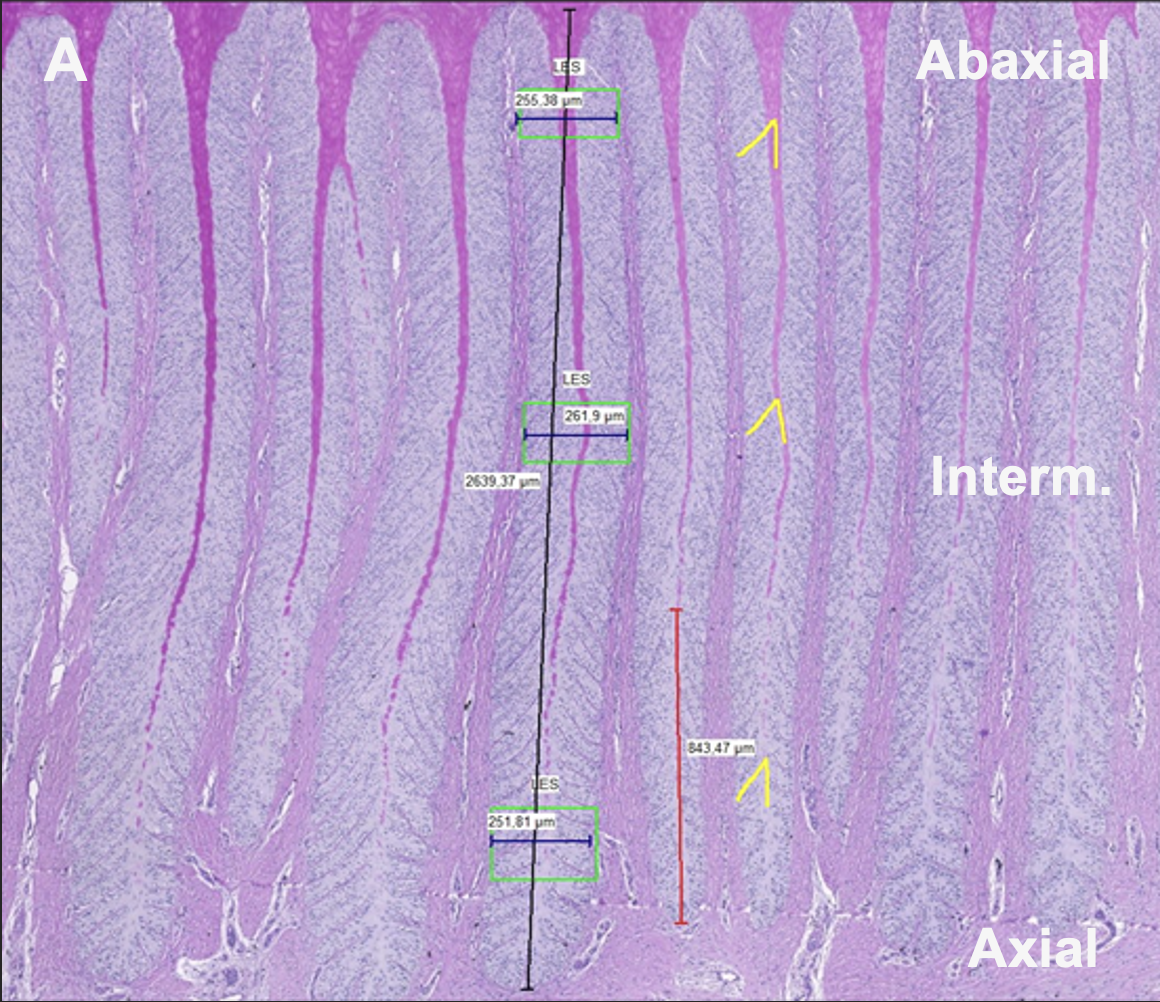

Supplement: S1 Data — (ZIP) [file pone.0286536.s001.zip › PEL histomorphometry.tif]

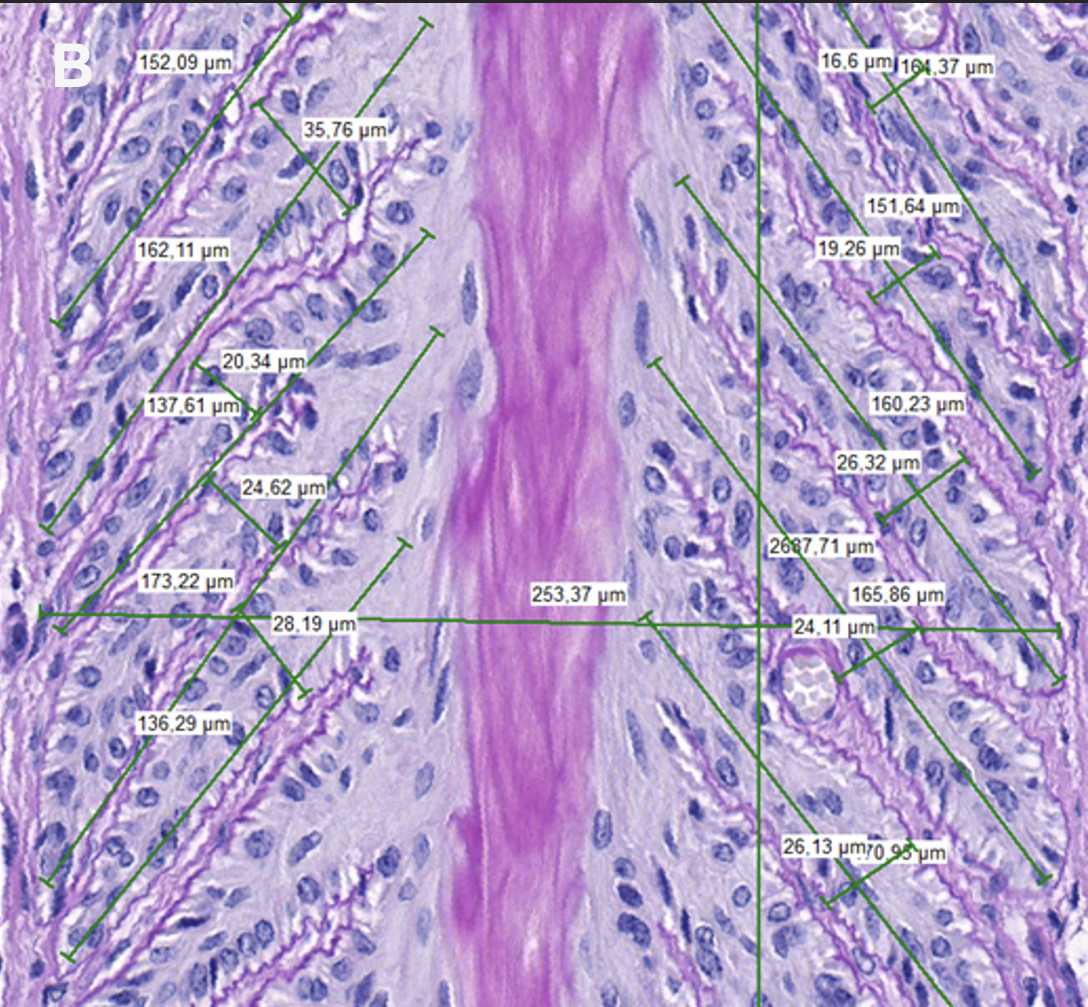

Supplement: S1 Data — (ZIP) [file pone.0286536.s001.zip › SEL histomorphometry.tif]

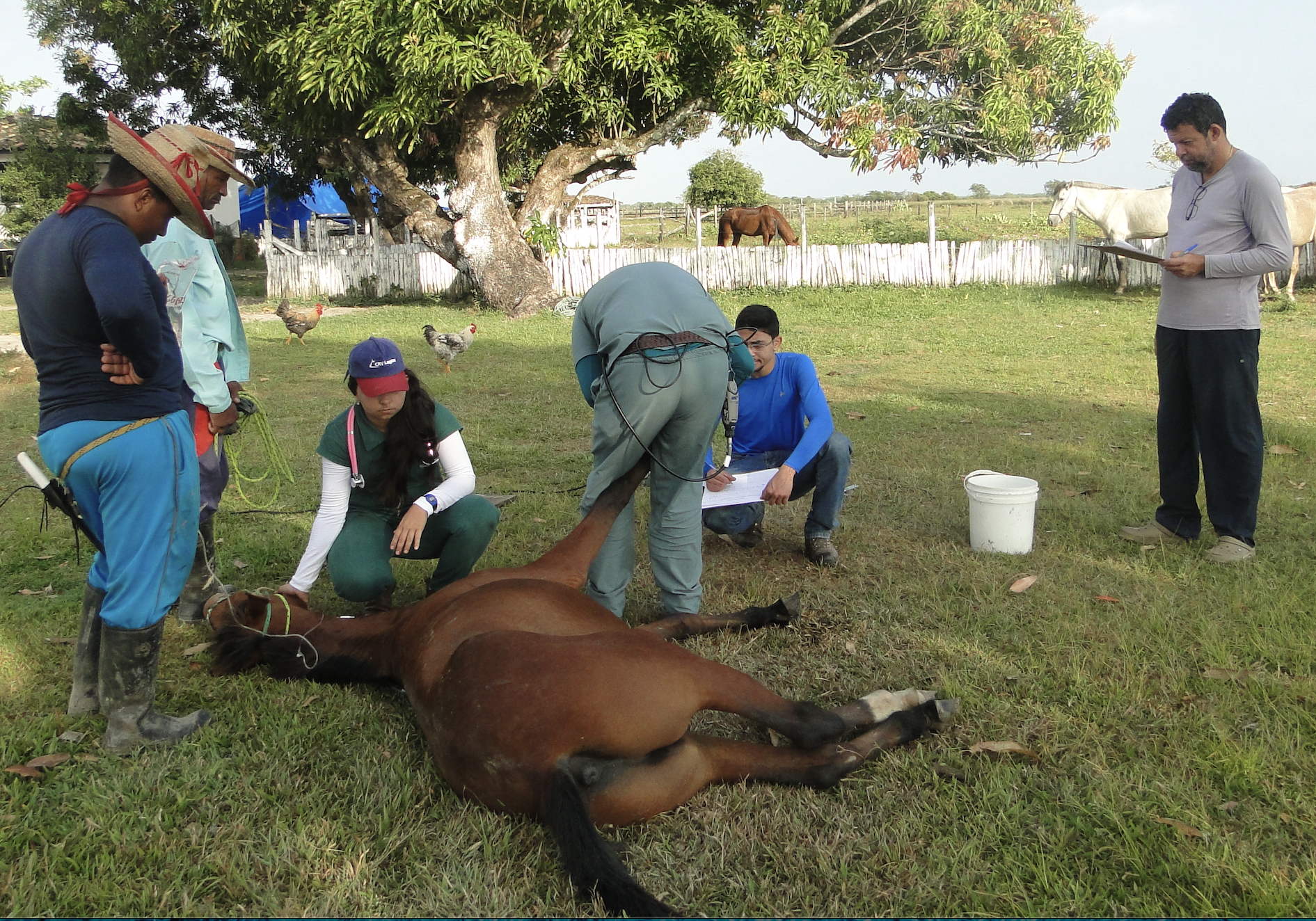

Supplement: S1 Data — (ZIP) [file pone.0286536.s001.zip › Under anesthesia.tif]
